# Premenstrual syndrome–related quality of life: associations with ultra-processed food consumption, mindful eating, well-being, and social media addiction in women

**DOI:** 10.1186/s12905-026-04267-8

**Published:** 2026-01-20

**Authors:** Fatma Elif Eroğlu, Büşra Açıkalın Göktürk, Neslihan Arslan, Fatma Kılıç

**Affiliations:** 1 Department of Nutrition and Dietetics, Gülhane Health Sciences Faculty, University of Health Sciences, Ankara, Turkey; 2 Department of Gastronomy and Culinary Arts, Faculty of Fine Arts, Design and Architecture, Ankara Medipol University, Ankara, Turkey; 3 Faculty of Health Sciences, Department of Nutrition and Dietetics, Bitlis Eren University, Bitlis, Turkey

**Keywords:** Premenstrual syndrome–related quality of life, Ultra-processed foods, Mindful eating, Well-being, Social media addiction

## Abstract

**Background:**

This cross-sectional study aimed to examine the associations between premenstrual syndrome–related quality of life (PMS-QoL), ultra-processed food (UPF) consumption, mindful eating, well-being, and social media addiction (SMA) among women.

**Methods:**

This study was conducted with 1,741 women aged 18–49 years (mean age: 24.86 ± 8.4). Data were collected via a web-based survey distributed through social media using snowball sampling. Premenstrual Syndrome Quality of Life Scale (PMS-QoL), Screening Questionnaire of Highly Processed Food Consumption (sQ-HPF), Mindful Eating Inventory (MEI), WHO-5 Well-Being Index (WHO-5), and Social Media Addiction Scale (SMAS) were used.

**Results:**

PMS-QoL was significantly correlated with social media addiction SMAS (r =-0.448,p < 0.001), sQ-HPF (r=-0.196, p < 0.001), and well-being (WHO-5)(r = 0.129, p < 0.001). MEI was positively correlated with WHO-5 (r = 0.179, p < 0.001) and age (r = 0.145, p < 0.001), and negatively correlated with SMAS (r = -0.100, p < 0.001), sQ-HPF (r =-0.086, p < 0.001), and BMI (r =-0.160, p < 0.001). In addition, sQ-HPF was positively correlated with SMAS (r = 0.208, p < 0.001).Linear regression analyses showed that PMS-QoL was significantly associated with BMI, sQ-HPF, SMAS, and WHO-5; (p < 0.001), while MEI was significantly associated with age, BMI, social media usage time, sQ-HPF, and WHO-5 (p < 0.001).

**Conclusion:**

This study highlights the multidimensional associations between PMS-related quality of life, eating behaviors, psychological well-being, and social media addiction. Higher UPF consumption and greater social media addiction were associated with poorer PMS-related quality of life, whereas mindful eating and higher well-being showed more favorable associations. From a women’s health perspective, these findings may point to the relevance of lifestyle-oriented and preventive approaches in relation to PMS-related quality of life.

## Introduction

Premenstrual syndrome (PMS) is a prevalent health concern characterised by a range of emotional, physical, and behavioural symptoms, with these symptoms typically abating with the onset of menstruation [1]. It is defined by the presence of at least one emotional, physical, or behavioural symptom that intensifies during the luteal phase preceding menstruation (1–2 weeks prior) and subsides with the onset of menstruation or within a few days [2]. Clinically significant PMS occurs in approximately 20–30% of women of reproductive age [3–5].According to Global Burden of Disease data, although the number of PMS cases increased by 46.5% between 1990 and 2019, the age-standardized global prevalence rate has remained stable (approximately 24/100000) [6].Meta-analyses of studies using self-reported retrospective data have found higher prevalence rates, reported as 48% in China [7] and 52% in Türkiye [8].A meta-analysis of studies with high methodological heterogeneity also found wide regional differences: 85% in Africa, 46% in Asia, and 40% in Europe [9].These differences in prevalence rates may be due to methodological differences such as sociocultural factors, symptom perception, diagnostic criteria, study design, and access to healthcare services [10].Moreover, the impact of symptoms associated with the menstrual period is frequently overlooked and underreported [11].

The exact pathophysiology of PMS is not yet fully understood and is thought to be related to hormonal fluctuations during the monthly cycle [12].Currently, the prevailing view is that symptoms arise from increased neurobiological sensitivity to fluctuations in ovarian hormones [13]. The absence of symptoms before menarche, during pregnancy, and after menopause, as well as the suppression of ovulation with GnRH agonists, support this mechanism [14]. Characterized by psychophysical symptoms such as emotional fluctuations, PMS symptoms begin during adolescence and can persist until menopause, leading to decreased functioning and a reduced quality of life [13, 14]. These results demonstrate that PMS is not only a cyclical condition but also a significant public health problem with an increasing global impact [15].

In addition to hormonal mechanisms, various biopsychosocial factors influencing the severity of PMS symptoms have been identified. In particular, psychiatric comorbidities such as anxiety and depression increase the risk and severity of PMS symptoms, which can complicate the diagnostic and treatment process [16]. The severity of PMS varies from person to person, and lifestyle factors, particularly eating behaviors, play an important role in managing symptoms. Therefore, eating behaviors can affect not only physiological processes but also hormonal balance, mental state, and appetite regulation, thereby determining the severity of PMS symptoms [17]. Appetite and food preferences can change during the phases of the menstrual cycle; the preference for fatty, sugary, and salty foods increases, particularly during the luteal phase [18]. These preferences, in conjunction with hormonal fluctuations, can lead to changes in eating behaviors and weight gain, particularly by facilitating overconsumption through the stimulation of dopamine-mediated reward mechanisms and negatively affecting appetite regulation [18, 19].In this context, changes in eating behaviors and food preferences are considered important factors shaping PMS symptom experiences through physiological and psychological processes [18]. When evaluated in terms of diet quality and pattern, diets rich in ultra-processed foods (UPFs) were positively associated with PMS, while traditional healthy diets rich in fruits and vegetables, whole grains, and nuts were found to have an inverse relationship [20].In addition to appetite changes related to the menstrual cycle, the increasing consumption of UPFs in recent years, coupled with developments in the food industry, has led to significant changes in individuals’ eating behaviors. UPFs are defined as industrially processed products with high energy density and containing additives and artificial ingredients [21]. Due to their high sugar, salt, and trans fat content, UPFs strongly stimulate the dopamine-mediated reward system, facilitating overconsumption and disrupting appetite regulation. They are associated with the risk of obesity, metabolic diseases, and cardiovascular disease [22, 23]. UPFs have also been associated with adverse reproductive health outcomes, hormonal imbalance, and mental health in women [24]. Moreover, increased UPF consumption has been linked to greater frequency and severity of PMS symptoms, including depression, anxiety, fatigue, irritability, and pain, as well as impairments in activities of daily living [25, 26]. These negative effects of UPF consumption also contribute to the decreased quality of life associated with PMS. However, some studies have not found a significant association between UPF consumption and menstrual irregularities and PMS-related symptoms [27], suggesting that further longitudinal studies are needed to clarify these associations. Finally, increased UPF consumption has been associated with digital media use, particularly exposure to food advertisements and distracting audiovisual stimuli, suggesting that screen-based environments, including social media, may indirectly influence eating behaviors and, consequently, PMS-related outcomes [28].

At this point, mindful eating emerges as a protective approach in supporting mental and physical health. Mindful eating refers to the individual focusing on hunger and fullness cues, staying present during the eating process, and consciously managing the eating experience [29]. Studies have shown that acquiring mindful eating behaviors can reduce emotional eating, overeating, and addictive eating behaviors [30]; mindful eating has been shown to be positively associated with healthy weight management, improved well-being, and PMS-related quality of life [31–33]. In this context, given the appetite fluctuations and increased emotional sensitivity during PMS, mindful eating is gaining importance as an approach to help regulate maladaptive eating behaviors and manage symptoms, thus preserving quality of life.

From a well-being perspective, a strong relationship has been observed between PMS symptoms and mental health. Evidence indicates that the tendency to seek psychological help increases as PMS severity increases in young individuals, and that the search for online mental support also becomes more common with increased symptoms [34, 35].

However, the factors affecting PMS symptoms are not limited to psychological factors; social media usage habits, which have become widespread with the rapid increase in digitalization, also play an important role by interacting with these processes. High levels of social media addiction (SMA) have been shown to be associated with the severity of PMS symptoms [36], and the same relationship was consistently observed in a study conducted on university students in Türkiye [37].

SMA is associated with distraction, stress, and anxiety, which directly affect women’s eating behaviors and mental health [38]. Evidence from recent studies indicates that SMA is associated with greater psychological distress (anxiety, stress, depression), which in turn is linked to maladaptive eating patterns-including reductions in mindful eating and increases in emotional/compulsive eating [39, 40].Increased emotional fluctuations during PMS, combined with anxiety and stress triggered by social media use, may contribute to unhealthy eating behaviors.Thus, excessive social media use may be associated with greater PMS symptom burden and poorer PMS-related quality of life. The current literature has generally addressed the relationships between PMS symptoms, eating behaviors, well-being, and SMA separately. However, studies that evaluate these variables together are quite limited, and research focusing specifically on PMS-related quality of life [18, 36] and UPF consumption remains scarce [40]. Therefore, this study aims to examine the relationships between PMS-related quality of life, UPF consumption, mindful eating, well-being, and SMA in adult women.

### Hypotheses

H1: Premenstrual syndrome–related quality of life is significantly associated with social media addiction, ultra-processed food consumption, and well-being.

- H1a: Higher levels of social media addiction are associated with lower premenstrual syndrome–related quality of life.
- H1b: Higher ultra-processed food consumption is associated with lower premenstrual syndrome–related quality of life.
- H1c: Higher well-being is associated with higher premenstrual syndrome–related quality of life.

H2: Mindful eating behaviors are significantly associated with social media addiction and ultra-processed food consumption.

- H2a: Higher levels of social media addiction are associated with lower mindful eating behaviors.
- H2b: Higher ultra-processed food consumption is associated with lower mindful eating behaviors.

## Methods

### Study design

This cross-sectional study was conducted between March and June 2025 and included 1741 adult women aged 18–49 years. The age range was chosen to align with the reproductive period during which PMS is most consistently observed and to minimise variability associated with perimenopausal transitions. At the beginning of the questionnaire, participants provided informed consent by selecting the statement “I voluntarily agree to participate in this study,” and only those who completed the entire survey were included. Participants were recruited using a non-probability, snowball sampling approach. Recruitment was initiated through WhatsApp and Gmail distribution lists, and the survey link was subsequently disseminated via participant-driven sharing within social networks. In online research settings, snowball sampling may overlap with features of convenience sampling due to this dissemination process.The survey obtained information on sociodemographic and lifestyle characteristics, including age, body weight, height, body mass index (BMI), marital status, education level, employment status, and economic status. Data were also collected on health-related behaviours such as nighttime and daytime sleep duration, social media usage time, and physical activity frequency. Reproductive characteristics included age at menarche (< 10, 11–12, 13–14, ≥ 15 years) and duration of menstruation (2–6 days, > 7 days). In accordance with ACOG guidelines indicating that most menstrual bleeding lasts 2–7 days, in this study we classified menstruation length as 2–6 days (normal) and ≥ 7 days (prolonged) [41].

**Fig. 1:**
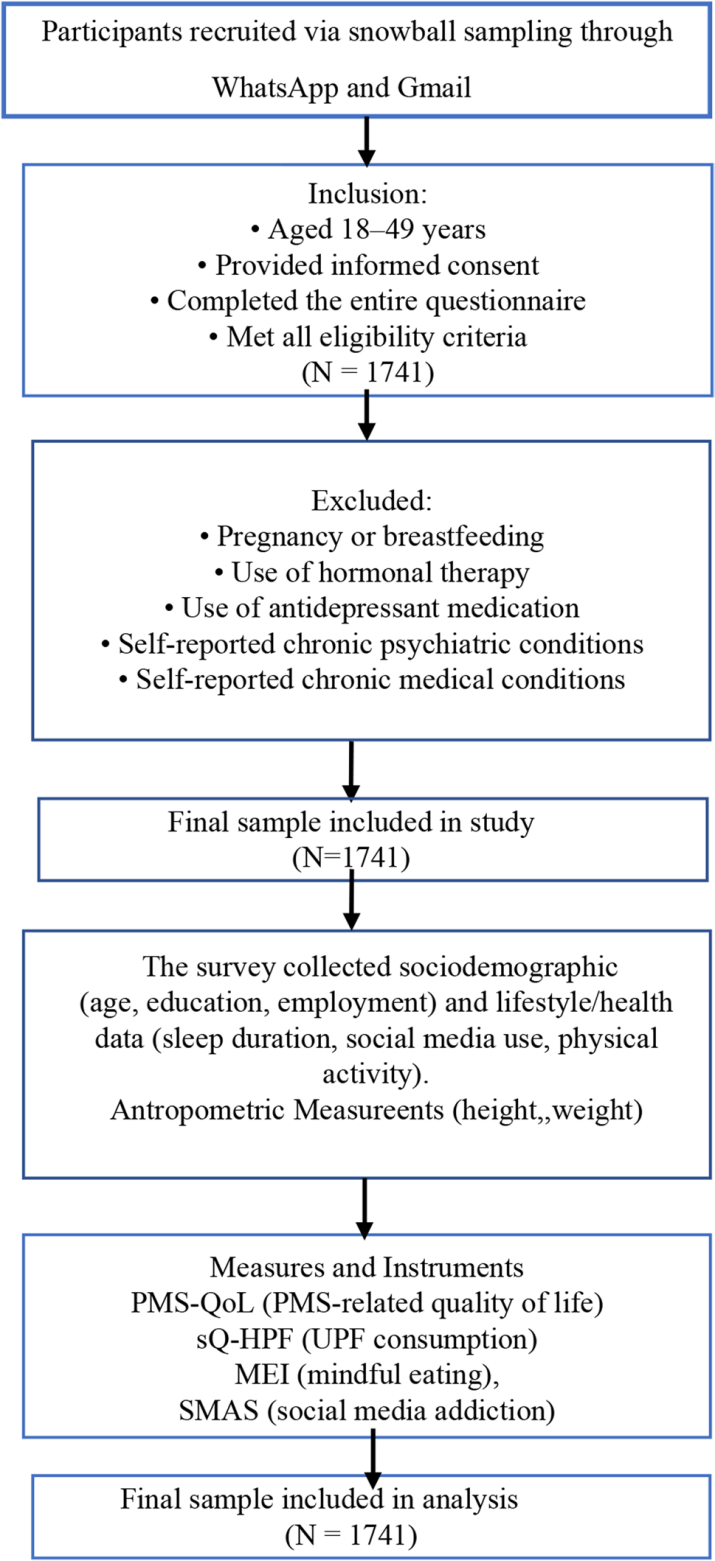
Flowchart of the study process

The exclusion criteria were assessed directly within the online survey. At the beginning of the questionnaire, participants were asked to self-report whether they were pregnant or breastfeeding, using hormonal therapy or antidepressant medication, or had any chronic psychiatric or medical conditions. These items were presented in a mandatory-response format following the informed consent. Participants who reported any of the exclusion criteria were excluded from the analysis based on self-reported information. Data were collected using a web-based survey that took approximately 20 min to complete. The Premenstrual Syndrome Quality of Life Scale (PMS-QoL) was used to measure the impact of PMS on quality of life. The Screening Questionnaire of Highly Processed Food Consumption (sQ-HPF) was used to assess ultra-processed food consumption. The Mindful Eating Inventory (MEI) was used to evaluate mindful eating behaviours. The WHO-5 Well-Being Index (WHO-5) was used to measure well-being. Finally, the Social Media Addiction Scale (SMAS) was used to assess social media addiction. A flowchart illustrating the study process is presented in Fig. 1. The figure summarizes the recruitment pathway, inclusion and exclusion criteria, reasons for exclusion, the final analytical sample (*n* = 1741), and the variables collected. It also lists the measurement tools used in the study, including the SMAS, sQ-HPF, MEI, PMS-QoL, and self-reported anthropometric indicators (Fig. 1).

### Participants

The mean age of the sample was 24.8 ± 8.4 years, and the mean BMI was 23.34 ± 3.72 kg/m². In terms of educational status, 62.8% had a bachelor’s degree, 29.2% had completed high school, 4.1% middle school, 2.2% primary school, and 1.8% held a master’s or doctoral degree. Most participants were single (79.7%) and unemployed (77.6%). Regarding economic status, 51.6% reported income equal to expenses, 37.1% below expenses, and 11.3% above expenses.

Physical activity levels varied within the sample: 47.8% reported no physical activity, while 17.7% exercised 1–2 times per week, 11.1% exercised 3–4 times per week, 4.7% exercised 5–6 times per week, 7.1% exercised daily, and the remainder reported less frequent activity.

Based on BMI categories, 72.7% were classified as normal weight, 17.3% overweight, 6.6% obese, and 3.4% underweight. The age at menarche most commonly ranged between 13 and 14 years (48.7%), followed by 11–12 years (30.4%), ≥ 15 years (18.0%), and < 10 years (2.9%). Menstrual duration was reported as 2–6 days by 68.8% of participants and > 7 days by 31.2% (Table 1).

**Table 1:** General characteristics of the individuals

Variables	X ± SD
Age(years)	24.8 **±** 8.4
BMI(kg/m^2^)	23.34 ± 3.72
	Number (%)
Education Level	
Primary	38(2.2)
Middle school	71(4.1)
High school	508(29.2)
Bachelor degree	1093(62.8)
Master/pHD degree	31(1.8)
Maritial status	
Married	353(20.3)
Single	1388(79.7)
Working status	
Yes	390(22.4)
No	1351 (77.6)
Economical status	
Income equals expenses	899(51.6)
Income exceeds expenses	196(11.3)
Income is less than expenses	646(37.1)
Physical activity status	
Everyday	124(7.1)
5–6 times a week	82(4.7)
3–4 times a week	193(11.1)
1–2 times a week	309(17.7)
Once every 15 days	93(5.3)
Once a month	108(6.2)
No	832(47.8)
BMI classification (kg/m^2^)	
Underweight	59 (3.4)
Normal weight	1265(72.7)
Overweight	302(17.3)
Obese (≥ 30)	115(6.6)
Mensturation age onset	
< 10 years	51(2.9)
11–12 years	529(30.4)
13–14 years	848(48.7)
> 15 years	313(18.0)
Menstruation length	
> 7 days	544(31.2)
2–6 days	1197(68.8)
Ultra processed food consumption	
High consumption	1103(63.4)
Low consumption	638 (36.6)

*BMI* Body Mass Index

### Anthropometric measurements

Anthropometric information (height and body weight) was obtained through self-reported data provided by the participants. Based on these self-reported measurements, BMI was calculated as weight (kg) divided by height squared (m²). BMI values were classified as underweight (< 18.50 kg/m²), normal weight (18.50–24.99 kg/m²), overweight (25.00–29.99 kg/m²), and obese (≥ 30.00 kg/m²) [42].

### Premenstrual syndrome quality of life scale (PMS-QoL)

The Premenstrual Syndrome Quality of Life Scale **(**PMS-QoL**)** was developed by Hadavi Bavili et al. to assess premenstrual syndrome. The scale consists of 22 reverse-coded items rated on a 5-point Likert scale. The total score ranges from 22 to 110, with higher scores indicating better quality of life and a reduced negative impact of PMS.The Cronbach’s α was 0.908 [43].The Cronbach’s α was found 0.925 in this sample.

### Screening questionnaire of highly processed food consumption (sQ-HPF)

The Screening Questionnaire of Highly Processed Food Consumption (sQ-HPF) was developed by Martínez-Pérez et al. [44] to assess highly processed food consumption, and its Turkish adaptation was carried out by Erdoğan Gövez et al. The total score ranges from 0 to 11, with scores of 6 or above indicating a high level of ultra-processed food consumption.The Cronbach’s α was 0.65 [45].The Cronbach’s α was found 0.768 in this sample.

### Mindful eating inventory (MEI)

The Mindful Eating Inventory (MEI) was developed by Peitz et al. to assess individuals’ mindful eating [46]. The Turkish adaptation was conducted by Bozkurt, Kocaadam et al. The Cronbach’s α was 0.86 [47]. The scale consists of 28 items rated on a 5-point Likert scale, with higher scores indicating greater awareness of eating behaviours. The Cronbach’s α was found 0.905 in this sample.

### WHO-5 well-being index (WHO-5)

The WHO-5 Well-Being Index was developed by the World Health Organization to measure well-being [48]. The Turkish validity and reliability study was conducted by Eser et al. [49]. The scale consists of 5 items rated on a 5-point Likert scale. Total scores range from 0 to 25, with higher scores indicating greater well-being.

### Social media addiction scale (SMAS)

The Social Media Addiction Scale (SMAS**)** was developed by Hazar to measure social media addiction. The scale consists of 23 items rated on a 5-point Likert scale, with higher scores reflecting greater levels of social media addiction The Cronbach’s α was 0.886 [50]. The Cronbach’s α was found 0.893 in this sample.

### Statistical analysis

The G*Power program was used for the preliminary power analysis of the PMS-QoL and the sQ-HPF. In a study design where the correlation between the PMS-QoL and the sQ-HPF was expected to be at the 0.14 level, the minimum required sample size, calculated with 80% power and an error coefficient of α = 0.05, was determined to be 850 individuals. All statistical analyses were conducted using IBM SPSS Statistics, version 24.0 (IBM Corp., Armonk, NY, USA). Normality of continuous variables was assessed using the Shapiro–Wilk test and probability–probability (P–P) plots. Variables that met the assumption of normal distribution were analyzed using independent samples t-tests, whereas non-normally distributed variables were compared using the Mann–Whitney U test. Based on the results, descriptive statistics were expressed as mean ± standard deviation for normally distributed variables, while categorical variables were summarised as frequencies and percentages. Pearson’s correlation coefficient was used for correlations between normally distributed continuous variables, while Spearman’s rank correlation coefficient was used for non-normally distributed variables. Linear regression analysis was conducted to assess the predictive relationship between the variables. Before interpreting the models, assumptions of linear regression were assessed. Normality of residuals was examined using histograms and Q–Q plots, homoscedasticity was assessed using residual-versus-predicted plots, and multicollinearity was evaluated using Variance Inflation Factors (VIF), with all VIF values < 5 and independence of errors was assessed using the Durbin–Watson statistic. Outliers were inspected using standardized residuals (>|3|). Cases with missing values were handled using listwise deletion. The total scores from the PMS-QoL, the MEI, and the sQ-HPF were each considered as dependent variables in separate regression models. Statistical significance was set at *p* < 0.05.

## Results

Descriptive characteristics of study variables were as follows: PMS-QoL (59.63 ± 15.99), MEI (4.46±0.38), SMAS (71.81 ± 3.67), sQ-HPF (6.56 ± 2.88), and WHO-5 (10.60 ± 5.52). Participants reported a mean daily social media usage time of 4.11 ± 2.00 h, an average daytime sleep duration of 1.36 ± 1.92 h, and a mean nighttime sleep duration of 7.18 ± 1.54 h. High UPF consumption was observed in 63.4% of the sample (*n* = 1103), while 36.6% (*n* = 638) reported low consumption (Table 2).

**Table 2:** Descriptive characteristics of the study variables

Variables	X ± SD
PMS-QoL score	59.63 ± 15.99
MEI score	4.46±0.38
SMAS score	71.81 ± 3.67
sQ-HPF score	6.56 ± 2.88
WHO-5 score	10.60 ± 5.52
Social media usage time(hours/day)	4.11 ± 2.00
Daytime sleep duration(hours/day)	1.36 ± 1.92
Night sleep duration(hours/day)	7.18 ± 1.54

*PMS-QoL* Premenstrual Syndrome Quality of Life Scale, *MEI *Mindful Eating Inventory, *SMAS* Social Media Addiction Scale, *WHO-5* WHO-5 Well-Being Index, *sQ-HPF *Screening Questionnaire of Highly Processed Food Consumption

As presented in Table 3, participants with higher sQ-HPF scores were significantly older (26.97 ± 0.40 vs. 23.70 ± 0.21 years, *p* < 0.001) and had lower PMS-QoL scores (57.90 ± 0.48 vs. 62.55 ± 0.62, *p* < 0.001) compared with those with lower scores. Participants with higher sQ-HPF scores also reported slightly lower MEI scores (4.45±0.37 vs. 4.50±0.40, 102.32 ± 0.50, *p* = 0.010) and higher SMAS scores (73.40 ± 0.41 vs. 69.10 ± 0.53, *p* < 0.001). In contrast, no significant differences were observed between groups in WHO-5 scores (10.48 ± 0.16 vs. 10.85 ± 0.22, *p* = 0.187) or daily social media usage time (4.28 ± 0.60 vs. 3.80 ± 0.77 h/day, *p* = 0.279).

**Table 3:** Evaluation of participants based on ultra-processed food consumption

Variables			Ultra-Processed Food Consumption					
			High consumption (*n* = 1103)		Low consumption (*n* = 638)	p value	T(df)	Cohen’s d
Age (years)			26.97 ± 0.40		23.70 ± 0.21	*p* < 0.001**	7.98 (1739)	0.39
PMS-QoL score			57.90 ± 0.48		62.55 ± 0.62	*p* < 0.001**	5.89 (1739)	0.29
MEI score			4.45±0.37		4.50±0.40	0.010^**^	2.59 (1739)	0.13
SMAS score			73.40 ± 0.41		69.10 ± 0.53	*p* < 0.001**	−6.28 (1739)	0.31
WHO-5 score			10.48 ± 0.16		10.85 ± 0.22	0.187	1.32 (1739)	0.07
Social media usage time (hours/day)			4.28 ± 0.60		3.80 ± 0.77	0.279	−1.08 (1739)	0.06

Independent samples t-test was used for between-group comparisons

*PMS-QoL* Premenstrual Syndrome Quality of Life Scale, *MEI* Mindful Eating Inventory, *SMAS* Social Media Addiction Scale, *WHO-5* WHO-5 Well-Being Index

*p *< 0.05**

As presented in Table 4, a significant correlation was observed between PMS-QoL and SMAS (*r*=−0.448, *p* < 0.001), and between social media usage time and SMAS (*r* = 0.257, *p* < 0.001). PMS-QoL was also negatively correlated with sQ-HPF (*r*=−0.196, *p* < 0.001) and positively correlated with WHO-5 (*r* = 0.129, *p* < 0.001). Age was negatively correlated with social media usage time (*r* =−0.296, *p* < 0.001), SMAS (*r*=−0.178, *p* < 0.001), and sQ-HPF (*r*=−0.155, *p* < 0.001), while positively correlated with BMI (*r* = 0.276, *p* < 0.001) and MEI (*r* = 0.145, *p* < 0.001). BMI was negatively correlated with MEI (*r* =−0.160, *p* < 0.001), social media usage time (*r*=−0.074, *p* = 0.002), and sQ-HPF (*r*=−0.055, *p* = 0.021). Social media usage time was negatively correlated with MEI (*r* =−0.136, *p* < 0.001) and PMS-QoL (*r* =−0.115, *p* < 0.001), and positively correlated with sQ-HPF (*r* = 0.144, *p* < 0.001). Additionally, sQ-HPF showed a positive correlation with SMAS (*r* = 0.208, *p* < 0.001) and a negative correlation with MEI (*r*=−0.086, *p* < 0.001). Finally, SMAS was negatively correlated with MEI (*r*=−0.100, *p* < 0.001), while WHO-5 was positively correlated with MEI (*r* = 0.179, *p* < 0.001).

**Table 4:** Correlation between variables

		Age (years)	BMI(kg/m^2^)	Social media usage time (hours/day)	PMS-QoL score	sQ-HPF score	SMAS score	WHO-5 score	MEI score
Age (years)	r								
	p	-							
BMI(kg/m^2^)	r	0.276^**^							
	p	< 0.001	-						
Social media usage time (hours/day)	r	−0.296^**^	−0.074^**^						
	p	< 0.001	0.002	-					
PMS-QoL score	r	0.029	0.024	−0.115^**^					
	p	0.221	0.327	< 0.001	-				
sQ-HPF score	r	−0.155^**^	−0.055^*^	0.144^**^	−0.196^**^				
	p	< 0.001	0.021	< 0.001	< 0.001	-			
SMAS score	r	−0.178^**^	−0.031	0.257^**^	−0.448^**^	0.208^**^			
	p	< 0.001	0.203	< 0.001	< 0.001	< 0.001	-		
WHO-5 score	r	−0.020	−0.041	−0.026	0.129^**^	−0.055^*^	−0.017		
	p	0.402	0.090	0.286	< 0.001	0.021	0.487	-	
MEI score	r	0.145^**^	−0.160^**^	−0.136^**^	0.045	−0.086^**^	−0.100^**^	0.179^**^	
	p	< 0.001	< 0.001	< 0.001	0.060	< 0.001	< 0.001	< 0.001	-

*PMS-QoL* Premenstrual Syndrome Quality of Life Scale, *sQ-HPF* Screening Questionnaireof Highly Processed Food Consumption, *SMAS* Social Media Addiction Scale, *WHO-5* WHO-5Well-Being Index , *MEI* Mindful Eating Inventory, *BMI* Body Mass Index

Spearman correlation *p *< 0.05**

**Fig. 2:**
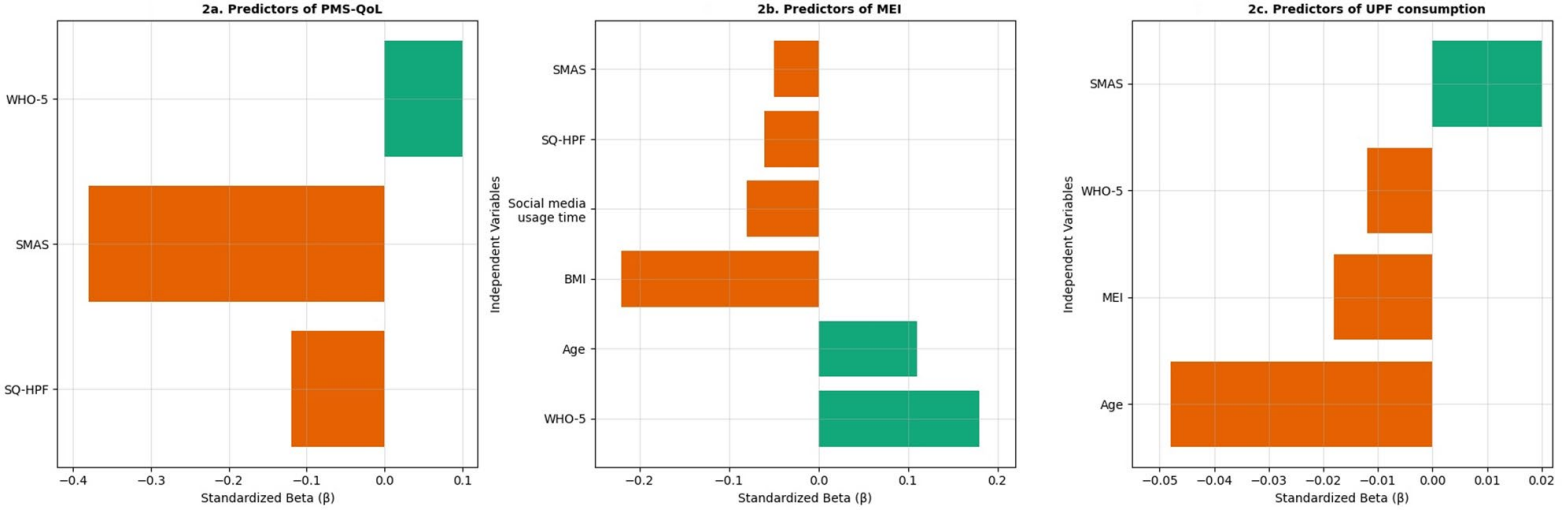
Fig 2. Standardized β coefficients from linear regression models predicting PMS-QoL (**a**), MEI (**b**), and UPF consumption (**c**). Abbreviations: *PMS-QoL *Premenstrual Syndrome Quality of Life Scale, *MEI* Mindful Eating Inventory, *sQ-HPF* Screening Questionnaire of Highly Processed Food Consumption, *SMAS* Social Media Addiction Scale, *WHO-5 *WHO-5 Well-Being Index, *BMI *Body Mass Index. *p < 0.05

Linear regression analysis was conducted to examine the variables associated with PMS-QoL. The overall model was statistically significant (R²=0.231, *p* < 0.001), accounting for 23.1% of the variance. Higher sQ-HPF scores were significantly associated with lower PMS-QoL (B=−0.631, β=−0.114, *p* < 0.001), and higher SMAS scores were also negatively associated with PMS-QoL (B =−0.506, β =−0.433, *p* < 0.001). In contrast, higher WHO-5 scores were positively associated with PMS-QoL (B = 0.305, β = 0.105, *p* < 0.001) (Table 5; Fig. 2a).

**Table 5:** Regression analysis for prediction premenstrual syndrome quality of life

Model	*Premenstrual Syndrome Quality of Life Scale score*							
	*Unstandardized B*		*Beta*	*t*	*p-value*	*Tolerance*		*VIF*
sQ-HPF score	−0.631		−0.114	−5.311	< 0.001		0.962	1.039
SMAS score	−0.506		−0.433	−20.199	< 0.001		0.966	1.036
WHO-5 score	0.305		0.105	4.996	< 0.001		0.996	1.004
			R^2^ = 0.231; p < 0.001*					

*Dependent variable: Premenstrual Syndrome Quality of Life Scale*

*sQ-HPF *Screening Questionnaire of Highly Processed Food Consumption,* SMAS *Social Media Addiction Scale,* WHO-5 *Well-Being Index

*p* < 0.05*

Linear regression analysis was conducted to examine variables associated with MEI. The model was statistically significant (R²=0.110, *p* < 0.001). Age (B = 0.148, β = 0.106, *p* < 0.001) and WHO-5 scores (B = 0.415, β = 0.194, *p* < 0.001) were positively associated with higher MEI scores. Conversely, BMI (B =−0.716, β=−0.226, *p* < 0.001), social media usage time (B =−0.537, β =−0.091, *p* < 0.001), sQ-HPF scores (B=−0.266, β =−0.065, *p* = 0.006), and SMAS scores (B=−0.063, β=−0.072, *p* = 0.002) were negatively associated with MEI scores (Table 6; Fig. 2b).

**Table 6:** Regression analysis of prediction mindful eating inventory

Model		Mindful Eating Inventory score							
		*Unstandardized B*		*Beta*		*t*	*p-value*	*Tolerance*	*VIF*
Age (years)		0.148		0.106		4.116	< 0.001	0.819	1.221
BMI (kg/m^2^)		−0.716		−0.226		−9.281	< 0.001	0.869	1.151
Social media usage time(hours/day)		−0.537		−0.091		−3.752	< 0.001	0.997	1.003
sQ-HPF score		−0.266		−0.065		−2.772	0.006	0.933	1.071
SMAS score		−0.063		−0.072		−3.043	0.002	0.941	1.063
WHO-5 score		0.415		0.194		8.537	< 0.001	0.995	1.005
				R^2^ = 0.110; p < 0.001*					

Linear regresion, Dependent variable; Mindful Eating Inventory

*sQ-HPF *Screening Questionnaire of Highly Processed Food Consumption, *SMAS *Social Media Addiction Scale, *WHO-5* WHO-5 Well-Being Index, *BMI* Body mass index

*p* < 0.05*

Linear regression analysis was conducted to examine variables associated with UPF consumption. The model was statistically significant (R²=0.228, *p* < 0.001). Age (β =−0.054, *p* < 0.001), MEI scores (β =−0.017, *p* = 0.004), and PMS-QoL scores (β = −0.024, *p* < 0.001) were negatively associated with sQ-HPF, whereas higher SMAS scores were positively associated with sQ-HPF (β = 0.018, *p* = 0.001) (Table 7; Fig. 2c).

**Table 7:** Regression analysis of prediction ultra-processed food consumption

Model		*Screening Questionnaire of Highly Processed Food Consumption score*								
		*Unstandardized B*		*Beta*	*t*		*p-value*	*Tolerance*	*VIF*	
Age (years)		−0.158	−0.054			−6.220	< 0 0.001	0.825	1.213	
BMI(kg/m^2^)		−0.004	−0.003			−0.150	0.881	0.826	1.211	
SMAS score		0.086	0.018			3.273	0.001	0.767	1.304	
PMS-QoL score		−0.133	−0.024			−5.096	< 0 0.001	0.781	1.280	
MEI score		−0.070	−0.017			−2.860	0.004	0.893	1.119	
WHO-5 score		−0.031	−0.016			−1.315	0.189	0.942	1.062	
			R^2^ = 0.228;p < 0.001*							

*PMS-QoL *Premenstrual Syndrome Quality of Life Scale,* MEI *Mindful Eating Inventory,* SMAS *Social Media Addiction Scale,* WHO-5 *Well-Being Index WHO-5, *BMI *Body mass Index, *Linear regresion*

Dependent variable: screening questionnaire of highly processed food Consumption,*p* < 0.05*

## Discussion

Premenstrual syndrome (PMS) is defined as a condition characterized by physical, behavioral, and emotional disturbances that occur during the luteal phase of the menstrual cycle and resolve with the onset of menstruation [51]. Sex hormones may affect the emotional state mechanism in women. It has been stated that emotional processing increases during the follicular phase and pre-ovulation phase, when estradiol concentrations are high and progesterone is low [13]. This indicates a model in which emotional perception increases depending on the stage of the menstrual cycle in women and that women’s emotions are affected by hormonal fluctuations [52].Hormonal fluctuations caused by the menstrual cycle may influence appetite mechanisms and eating behavior. Therefore, the presence of PMS has been associated with changes in the consumption of certain foods [53]. This study examines the relationship between PMS quality of life, UPF consumption, and mindful eating. In line with the between-group comparisons, participants with higher ultra-processed food consumption exhibited lower PMS-related quality of life and higher social media addiction scores, whereas no significant group differences were observed for well-being or daily social media usage time.The results of the study indicate that higher UPF consumption is associated with lower PMS-realated quality of life.However, it was found that individuals with high mindful eating scores tended to have higher PMS quality of life scores. This finding may indicate that the severity of PMS is related to dietary habits, while PMS symptoms themselves may also contribute to food choices by altering appetite regulation and cravings [18]. A study examining the effect of PMS on food choices indicated that women consumed more UPFs, such as cakes, pastries, and sweets, which are high in simple sugars, and consumed less milk, vegetables, and fruit [54]. This situation can be explained by estrogen and progesterone affecting brain anatomy during the premenstrual period, thereby shaping food choices [55].There are several hypotheses regarding how estrogen and progesterone contribute to brain anatomy and function. The main hypothesis suggests that estrogens have an effect on serotonin and dopamine neurotransmitters and mitochondrial function [56]. It is also known that hormonal fluctuations during the premenstrual period can cause temporary mood changes such as sadness, anxiety, and low mood. A study conducted on young adults suggested that an increase in UPF consumption may exacerbate conditions such as depression, anxiety, fatigue, irritability, and pain, thereby potentially reducing PMS quality of life [25]. In our study, it was found that 63.4% of participants consumed UPF. This proportion may indicate that UPF intake is a common dietary behavior among women of reproductive age. These findings underline the potential importance of the food environment and dietary awareness in reproductive health strategies.Evidence also points to the possibility that a Western-style diet and UPF snacks might help alleviate PMS symptoms and thereby improve PMS quality of life [57]. Although our findings did not support this, reporting such inconsistencies in the literature is important to highlight potential differences between populations and methodologies. Similarly, one study observed a negative relationship between a diet rich in processed meat, fast food, vegetable oil, mayonnaise, fried foods, salty snacks, refined grains, and simple sugars and PMS quality of life [58]. In addition, one study reported that increased consumption of sweet foods, fast food, fried foods, coffee, and alcohol was associated with the severity of PMS symptoms, while increased consumption of fruits and vegetables, low-fat, and high-fiber foods was associated with a reduction in symptoms [59]. A study conducted in Korea among women aged 20–49 suggested that high consumption of UPF snacks may increase the risk of PMS and reduce quality of life [27]. Another study found no relationship between the consumption of foods containing UPF, which are high in fat and carbohydrates, and PMS quality of life [60]. Another study suggested that excessive consumption of highly processed foods containing high levels of simple carbohydrates, fat, and salt, as well as alcohol consumption, may contribute to an increase in the severity of PMS symptoms, while healthy food choices may help improve PMS quality of life and reduce symptom severity [61]. Taken together, these findings highlight that dietary patterns may play a dual role in PMS, acting as both potential risk and protective factors depending on food type and quality. Likewise, one study reported that consuming healthy foods and practicing mindful eating may positively influence PMS quality of life by improving overall well-being and functionality [62]. In contrast, a randomized controlled trial comparing groups that received dietary counseling with those that did not found no significant relationship between healthy eating habits and PMS quality of life [63]. Another study reported no significant difference in dietary patterns and food choices between women with PMS and a control group [18]. These inconsistencies may suggest that the effects of dietary behaviors on PMS could vary depending on study population, methodology, and cultural context. Importantly, this underlines the need for more standardized measures in future PMS and nutrition research. While several studies support the associations observed in this study, some investigations have reported weaker or inconsistent relationships, indicating that these patterns may vary across populations and methodological approaches. In our study, a negative association was observed between the total PMS-QoL score and UPF consumption. Higher UPF consumption was associated with lower PMS-QoL scores. Given that higher PMS-QoL scores represent better quality of life, the observed negative associations indicate a decrease in PMS-related quality of life.However, no significant relationship was found between the PMS-QoL score and MEI scores. Additionally, a positive and significant relationship was found with age and MEI scores. This may suggest that with increasing age, there is a greater tendency to score higher on mindful eating, possibly due to increased health awareness and life experience. Similar to the findings of our study, one investigation emphasized that lower total fat, saturated fatty acid, and cholesterol intake in adults with PMS compared to young people was positively related to MEI scores [20].

Increased energy intake and consumption of highly processed foods may be associated with an increased risk of obesity and a decrease in PMS quality of life [64]. It has been observed that emotional eating behavior tends to increase and mindful eating behavior appears to decrease with the increase in PMS. This mechanism may reflect that PMS symptoms, through hormonal and emotional changes, increase the tendency for high-energy food consumption while reducing mindful eating behaviors. Dopaminergic signaling represents another potential pathway through which estrogen and progesterone may influence mood regulation. Dopamine plays a key role in reward processing, pleasure, motivation, decision-making, motor control, and learning, and is therefore commonly referred to as the “reward neurotransmitter” [65]. Previous evidence indicates that unhealthy eating behaviors, characterized by increased consumption of high-energy and high-fat foods, are associated with emotional eating, reduced mindful eating, and the development of obesity [66]. Similar to the literature, our study found a weak negative correlation between BMI and MEI scores. Unlike some previous studies, a weak negative association was also observed between BMI and UPF consumption; however, the small effect size indicates limited practical importance. This may be due to the fact that the participants in our study were generally individuals of ideal weight. This suggests that sample characteristics, such as BMI distribution, could influence the observed associations. Obese/overweight individuals have been found to consume more UPFs and have higher levels of food craving and unconscious eating behavior awareness. Conversely, one study reported no significant relationship between individuals’ PMS quality of life total scores and food consumption and BMI [67].

Social media addiction (SMA) is thought to have a negative impact on an individual’s psychosocial well-being and overall happiness. It has been associated with various physical, psychological, and mental health problems such as anxiety, stress, depression, emotional exhaustion, low self-esteem, and poor sleep quality [68]. Some evidence suggests that SMA may exacerbate the effects of PMS due to its negative impact on sleep quality, mental and physical health [69]. A study conducted on young adults found that SMA may affect PMS symptoms and that as SMA increases, the average scores for depressive thoughts, anxiety, irritability, and depressive mood subdimensions also increase [37]. Another study indicated a statistically significant correlation between increasing SMA and higher PMS incidence [36]. Another study noted that internet addiction may be associated with menstrual pain severity and impairment in social life [70]. In another randomized controlled trial, the experimental group received social media-based support via a smartphone app, text messages, and emails throughout one menstrual cycle. It was reported that women with PMS experienced a reduction in premenstrual symptoms and an increase in physical activity compared to the control group, and that this was positively associated with an improvement in PMS quality of life [71]. Similarly, one study indicated that social media-based cognitive-behavioral therapy administered over 8 weeks reduced the severity of PMS symptoms and improved PMS quality of life [72]. In our study, social media usage time and total SMAS score were negatively correlated with the PMS-QoL scores. Furthermore, social media usage time was negatively correlated with MEI and PMS-QoL scores, but positively correlated with SMAS and UPF. This pattern may indicate that increased time spent on social media could contribute to higher SMA, which in turn may negatively influence mindful eating behaviors and increase the consumption of UPFs. Consistent with this finding, one study reported that those with high digital media usage also consumed more UPFs [73]. Another study reported that as time spent online increases, excessive food consumption becomes more common, and the foods consumed are generally UPFs [74]. These findings highlight that digital behaviors and lifestyle patterns should not be overlooked in the context of PMS and nutrition research.

It is well established that women experiencing PMS may also be affected by psychological problems related to the syndrome [75]. For example, one study indicated that the most common psychological factors accompanying PMS are irritability and anger levels [76]. In line with this, one study indicated a negative relationship between PMS and mental well-being [77]. Another study reported that PMS may increase anxiety, depression, and stress levels [78]. One investigation found that as PMS scale scores increased, and mental well-being scores decreased [79]. Likewise, one study emphasized that individuals may feel more depressed and tired, and their levels of mental well-being may decrease due to the decline in quality of life associated with PMS [80]. In our study, PMS-QoL was also found to be positively associated with WHO-5. This suggests that improvements in quality of life during the premenstrual period could be linked to better mental well-being outcomes. Supporting this, one study similarly indicated that as PMS-related quality of life increased, perceived stress levels decreased and mental well-being increased [75]. Together, these findings highlight the potential importance of holistic approaches that integrate nutritional, psychological, and lifestyle factors when addressing PMS-related quality of life. Therefore, the findings should be interpreted as hypothesis-generating rather than confirmatory.

### Limitations and strengths

This study has several strengths. It is one of the few studies to examine PMS-related quality of life in relation to UPF consumption, mindful eating, and SMA within a large and diverse sample of women. The inclusion of participants from a wide age range and different socioeconomic backgrounds increases the representativeness and robustness of the findings. Additionally, the study employed validated and widely used measurement tools (PMS-QoL, MEI, SMAS, WHO-5, and sQ-HPF), which strengthens the reliability and comparability of the results.

Given the cross-sectional design, the relationships observed in this study should be interpreted as associations rather than causal effects, and reverse causation cannot be ruled out; therefore, causal inference cannot be established. In addition, all data-including anthropometric indicators such as BMI-were self-reported, which may introduce recall or reporting bias. Because height and weight were self-reported, BMI values may be affected by reporting bias, and no subsample validation was conducted to assess measurement accuracy; however, previous validation studies in adult women have demonstrated a strong correlation between self-reported and measured BMI, with a tendency toward slight underestimation at higher BMI levels [81]. Nevertheless, some limitations should be acknowledged. In addition, the snowball sampling approach used in this study-conducted entirely through online platforms such as WhatsApp and Gmail may have introduced selection bias, potentially leading to an overrepresentation of individuals who are more digitally active or belong to certain socioeconomic groups, predominantly from urban settings, which may further limit generalizability to rural populations or individuals with limited internet access.Snowball sampling through digital platforms may have resulted in geographic clustering, which could further limit the generalizability of the findings. Additionally, several potential confounders, such as psychiatric comorbidities, medication use, dietary restraint, sleep quality, and menstrual irregularities-were not assessed, and the exclusion of psychiatric disorders relied solely on self-report without screening instruments, which may influence both PMS and eating behaviors; therefore, the findings should be interpreted accordingly. In addition, PMS symptoms may fluctuate across the menstrual cycle, and data were collected at a single time point without verifying menstrual phase, which may influence symptom variability. Moreover, the limited variability in BMI and the high proportion of physically inactive participants may further restrict the generalizability of the findings. Although the instruments used in this study (PMS-QoL, MEI, sQ-HPF, and SMAS) have been previously validated in Turkish populations, subtle cultural nuances in item interpretation may still exist and should be considered when interpreting the findings.Although the regression models accounted for a meaningful proportion of variance in PMS-related quality of life (R² = 0.231), a considerable proportion remained unexplained, indicating the potential influence of other unmeasured factors. Finally, as the study was conducted in a single country, cultural and contextual factors may limit the generalizability of the results to other populations.

Future longitudinal and interventional studies should use the PMS-QoL total score as the primary endpoint, with a clinically meaningful improvement defined as at least a 30% reduction from baseline. This threshold will enable reproducible sample-size calculations and facilitate comparison across future trials.

## Conclusion

Premenstrual syndrome is a multidimensional condition associated with women’s eating behaviors, food choices, well-being, and overall quality of life. In this study, higher consumption of ultra-processed foods and greater social media addiction were associated with poorer PMS-related quality of life, whereas higher mindful eating and well-being were associated with more favorable outcomes. These findings reflect correlational, not causal, relationships; therefore, no conclusions regarding causality or directionality can be drawn. While the present findings highlight potential associations between dietary behaviors, social media use, and PMS-related quality of life, these observations should be interpreted within a hypothesis-generating framework. Further longitudinal and interventional studies are needed before such associations can inform clinical screening approaches or public health strategies.

By examining nutrition, mindful eating, and social media use together, this study provides a holistic perspective on factors linked to PMS-related quality of life.

## Data Availability

Data will be available upon request.

## Abbreviations

BMI: Body Mass Index
MEI: Mindful Eating Inventory
PMS: Premenstrual Syndrome
PMS-QoL: Premenstrual Syndrome Quality of Life Scale
SMA: Social Media Addiction
SMAS: Social Media Addiction Scale
sQ-HPF: Screening Questionnaire of Highly Processed Food Consumption
UPF: Ultra-Processed Food
WHO-5: WHO-5 Well-Being Index
